# Prediction of Freezing of Gait in Parkinson’s Disease Using Wearables and Machine Learning

**DOI:** 10.3390/s21020614

**Published:** 2021-01-17

**Authors:** Luigi Borzì, Ivan Mazzetta, Alessandro Zampogna, Antonio Suppa, Gabriella Olmo, Fernanda Irrera

**Affiliations:** 1 Department of Control and Computer Engineering, Politecnico di Torino, 10129 Turin, Italy; gabriella.olmo@polito.it; 2 Department of Information Engineering, Electronics and Telecommunication, Sapienza University of Rome, 00184 Rome, Italy; ivan.mazzetta@uniroma1.it (I.M.); fernanda.irrera@uniroma1.it (F.I.); 3 Department of Human Neurosciences, Sapienza University of Rome, 00185 Rome, Italy; alessandro.zampogna@uniroma1.it (A.Z.); antonio.suppa@uniroma1.it (A.S.); 4 IRCCS NEUROMED Institute, 86077 Pozzilli, Italy

**Keywords:** wearable sensors, machine learning, freezing of gait (FOG), FOG prediction, levodopa, Parkinson’s disease, degradation of gait pattern

## Abstract

Freezing of gait (FOG) is one of the most troublesome symptoms of Parkinson’s disease, affecting more than 50% of patients in advanced stages of the disease. Wearable technology has been widely used for its automatic detection, and some papers have been recently published in the direction of its prediction. Such predictions may be used for the administration of cues, in order to prevent the occurrence of gait freezing. The aim of the present study was to propose a wearable system able to catch the typical degradation of the walking pattern preceding FOG episodes, to achieve reliable FOG prediction using machine learning algorithms and verify whether dopaminergic therapy affects the ability of our system to detect and predict FOG. Methods: A cohort of 11 Parkinson’s disease patients receiving (on) and not receiving (off) dopaminergic therapy was equipped with two inertial sensors placed on each shin, and asked to perform a timed up and go test. We performed a step-to-step segmentation of the angular velocity signals and subsequent feature extraction from both time and frequency domains. We employed a wrapper approach for feature selection and optimized different machine learning classifiers in order to catch FOG and pre-FOG episodes. Results: The implemented FOG detection algorithm achieved excellent performance in a leave-one-subject-out validation, in patients both on and off therapy. As for pre-FOG detection, the implemented classification algorithm achieved 84.1% (85.5%) sensitivity, 85.9% (86.3%) specificity and 85.5% (86.1%) accuracy in leave-one-subject-out validation, in patients on (off) therapy. When the classification model was trained with data from patients on (off) and tested on patients off (on), we found 84.0% (56.6%) sensitivity, 88.3% (92.5%) specificity and 87.4% (86.3%) accuracy. Conclusions: Machine learning models are capable of predicting FOG before its actual occurrence with adequate accuracy. The dopaminergic therapy affects pre-FOG gait patterns, thereby influencing the algorithm’s effectiveness.

## 1. Introduction

Freezing of gait is a form of paroxysmal akinesia (i.e., loss of movement) affecting gait in more than 50% of patients with Parkinson’s disease (PD) [1]. Freezing of gait (FOG) is defined as a “brief, episodic absence or marked reduction of forward progression of the feet despite having the intention to walk” [2]. FOG is rather heterogeneous in terms of clinical phenomenology (i.e., shuffling steps, trembling legs or complete akinesia) [3]; duration of a single episode (with half of episodes lasting less than 5 s and 90% less than 20 s) [4]; and triggering factors, including environmental circumstances (e.g., turning, gait initiation, narrow spaces) [3,5,6], cognitive challenges (e.g., dual tasking) [7] and emotional stress (e.g., anxiety) [8]. FOG represents one of the most challenging and disabling symptoms in PD [2,9,10] since it increases the risk of falls [7,11] and is an early predictor of shortened survival [12]. Previous experimental studies with gait analysis have demonstrated that besides FOG episodes, patients with FOG are characterized by abnormal spatial–temporal gait parameters, such as slower and shorter stride lengths, greater spatial and temporal stride-to-stride variability and higher asymmetry between the two legs’ mobility than patients without FOG [13,14]. Specific spatial–temporal gait parameters (e.g., step to step amplitude and variability) degrade progressively up to the occurrence of FOG, raising the opportunity to recognize typical pre-FOG periods [15], intended as specific movement patterns occurring during effective gait just before FOG episodes. The recognition of pre-FOG periods would allow the adoption of corrective strategies to prevent or overcome FOG, such as the administration of external sensory queuing [16].

The automatic detection of FOG episodes has been widely explored in the last 15 years, making use of wearable sensors [17]. Wearable sensors are cheap, lightweight and unobtrusive, thereby representing a feasible solution for objectively evaluating FOG both in the laboratory and in the home [18]. The employed sensors include commercial [19] or prototype inertial measurement units [20], smartphones [21,22] and single accelerometers and/or gyroscopes [18,23], in combination with surface electromyography [24]. Different locations have been explored for sensor positioning, including waist, shin, thigh, foot and chest [25]. Most experimental protocols are carried out in laboratory settings [26], and include a set of activities such as walking, turning, timed up and go (TUG) test [27] and simulated activities of daily living (ADL) [19,28].

Inertial wearable sensors also enable monitoring of spatial–temporal gait degradation in patients with FOG, possibly useful for the recognition of pre-FOG periods. Indeed, their usage, in combination with machine learning (ML) analysis, has recently smoothed the path for FOG prediction [29,30,31,32,33,34]. By examining several time and frequency-domain gait features, these studies have achieved the real-time detection of pre-FOG periods [29,31,32,35]. However, the reported performance of the ML analysis in the prediction of FOG is suboptimal in terms of accuracy, possibly reflecting the clinical heterogeneity of the cohorts under investigation. For instance, the accuracy in the prediction of FOG in PD would benefit from evaluating the effect of levodopa (L-dopa), which is known to improve spatial–temporal gait parameters (e.g., step length and velocity) [13]. Accordingly, the condition of the patient with respect to the dopaminergic therapy would affect the algorithm’s effectiveness, and thus the accuracy of FOG prediction. None of the previous studies using inertial wearable sensors and ML analysis to predict FOG has assessed and compared patients receiving (on) and not receiving (off) dopaminergic therapy.

In this study, we used inertial wearable sensors and ML analysis to predict FOG by detecting pre-FOG periods in patients with PD. Moreover, we compared the ML prediction’s effectiveness in patients on and off therapy, which had not been done in previous studies, in order to assess whether dopaminergic therapy alters the gait patterns in the very few seconds preceding the onset of a FOG episode. Shedding light on the role of the on/off patient condition would help to optimize ML approaches and improve the prediction of FOG by using ecological algorithms regardless the patient’s clinical state. Differently from the previous works, mostly employing all components from 3-axial accelerometers, we employed a single angular velocity signal from sensors placed on shins, in order to make the algorithm as interpretable as possible. Furthermore, we implemented a step-to-step segmentation process and extracted features from each step. Finally, we made no assumptions regarding the time frame in which the degradation manifests, thereby performing the analysis while employing different durations of the pre-FOG window.

The rest of this paper is organized as follows. In Section 2.1 the patients’ enrollment procedure is described, along with demographic and clinically relevant data. In Section 2.2, the experimental set-up and data acquisition procedures are explained. Section 2.3 reports the data pre-processing details and a discussion on the extracted features, whereas in Section 2.4 and Section 2.5 the algorithms for FOG and pre-FOG detection are described. Section 2.6 provides descriptions of the performance evaluation metrics used in this study. The obtained results are reported and discussed in Section 3 and Section 4 respectively, whereas in Section 5 conclusions are drawn.

## 2. Materials and Methods

### 2.1. Subjects

We enrolled eleven patients with PD and FOG from the Movement Disorder outpatient clinic of the Department of Human Neurosciences, Sapienza University of Rome, Italy. We included patients according to the following clinical criteria: diagnosis of idiopathic PD based on current consensus criteria [36]; lack of dementia (Mini-Mental State Examination—MMSE > 24); presence of FOG directly verified by physical examination of two neurologists, experts in movement disorders; ability to walk independently; lack of comorbidities possibly affecting gait (e.g., neuropathies, rheumatic and orthopaedic disorders). To assess patients’ motor, cognitive and emotional functions, the clinical examination included the following standardized scales and scores: the Hoehn and Yahr scale (H&Y), the Movement Disorder Society—unified Parkinson’s disease rating scale (MDS-UPDRS) part III (modified), FOG questionnaire (FOG-Q), MMSE, frontal assessment battery (FAB), Hamilton depression rating scale (HAM-D) and Beck anxiety inventory (BAI). During the experimental sessions, we studied patients both receiving (1 h after L-dopa intake) and not receiving (after L-dopa withdrawal for at least 12 h) dopaminergic therapy (i.e., on and off state of therapy, respectively). Finally, we calculated the L-dopa equivalent daily doses (LEDDs) for each patient [37]. Table 1 and Table 2 summarize the demographic and clinical features of patients with PD and FOG enrolled in this study. In agreement with the Declaration of Helsinki, the experimental procedures were approved by the institutional review board of Sapienza University of Rome, Italy. Additionally, all the patients gave written informed consent to experimental procedures.

### 2.2. Experimental Procedures and Data Acquisition

**Figure 1:**
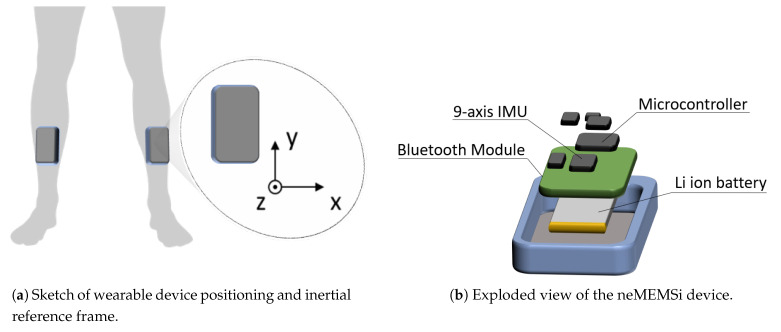
Sensor positioning and composition.

The motor task consisted of 7 m TUG test requiring patients to get up from a chair, walk in a straight line for 7 m, turning, walking back and sitting down. To increase the occurrence of FOG episodes, the TUG test was performed in a free living-like environment implying factors that commonly elicit FOG in a domestic setting. More in detail, the TUG test implied the passage from a spacious room to a narrow and furnished corridor (about 1.5 m wide) with the interposition of an open door [14]. During TUG tests, PD patients were video-recorded through a camera and monitored by two Inertial Measurement Units (IMUs) placed and fixed on the shins (Figure 1a) through elastic bands, which allowed a good and permanent adhesion during the tests. Video-recordings were used for the offline clinical assessment by two independent neurologists, experts in movement disorders, serving as gold standard evaluation for FOG detection. More in detail, two independent neurologists separately identified the start and end of FOG episodes and, in case of discrepancy, performed a common assessment to resolve the ambiguity. The IMUs positioning on the patient is implemented so that when the patient is standing the y-axis represents the inverse gravity vector and x-axis lies in the frontal plane. Hence, the angular velocity around the x-axis enables a good representation of the human motion during linear gait. The STMicroelectronics system-on-board prototypes neMEMSi [38] were equipped with: a 9 axis IMU (LSM9DS0), integrating a ±16 g 3D accelerometer, a ±12 Gauss 3D magnetometer and a ±2000 dps 3D gyroscope; a Bluetooth V3.0 module (BT33); a lithium-ion battery; an ultralow-power 32-bit microcontroller (STM32L1) (Figure 1b). Additionally, neMEMSi included a temperature sensor, a hygrometer sensor and a pressure sensor that were not used for this study. We performed preliminary conventional calibration of the inertial sensors. It consisted in a software correction of the displacement of the IMUs framework respect to the earth framework, before their positioning on the patient.

Real-time IMU data were acquired with a sampling frequency of 60 Hz, acceleration full scale of ±2 g, angular velocity full scale of ±245 dps. No additional analog/digital filter was added respect to the ones specified in the datasheets. The resulting data were sent in real-time to a personal computer through the neMEMSi Bluetooth module and progressively saved in CSV format.

**Figure 2:**
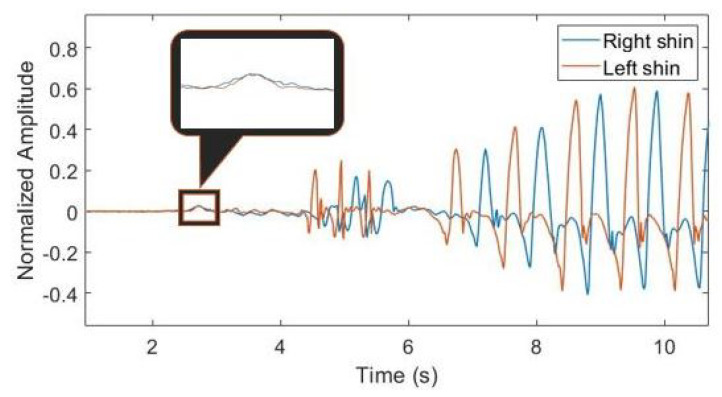
Normalized angular velocity around the x axis versus time for the two shins during the transition from sitting to standing up.

Each CSV file was related to a single test. Data in CSV files were processed offline, as described in the next section. For the synchronization of the two devices, data collection starts when the patient is sitting down. When the patient stands up, an evident peak in the collected data from the three axis gyroscopes takes place, as can be observed in Figure 2 for the x-axis of the gyroscope. In that plot the normalized angular velocity around the x axis is drawn versus time. At time t = 2.6 s the patient stood up, and a peak from each device is present. In the following few seconds (until t = 6 s) data are not meaningful because the patient was arranging her/his position. After t = 6.5 s the patient started walking. By superimposing the standing-up peaks related to the two legs, the relative delay from each other can be calculated. The mentioned method allows a perfect synchronization between signals from the two shins, which conserve their phase shift along the whole test duration.

### 2.3. Preprocessing

In this study we employed a single component of the angular velocity signal, which describes the principal angular movement of the leg. Specifically, the angular velocity signal around the x-axis is addressed (see Figure 1a), in the following reported as $\omega_{x}$. As a first operation, we normalized the raw input data $\omega_{x}$, employing the mean-normalization formula reported in Equation (1). It consists of removing the data mean value and dividing it by the data range; hence, the obtained normalized data are zero-mean and unit range. Standardization allows us to process a homogeneous range of motion data for the entire population, and to perform data segmentation in a subject-independent way.

(1) $$\omega_{x}^{\prime }=\frac{\omega_{x}-mean\left(\omega_{x}\right)}{max\left(\omega_{x}\right)-min\left(\omega_{x}\right)}$$

**Figure 3:**
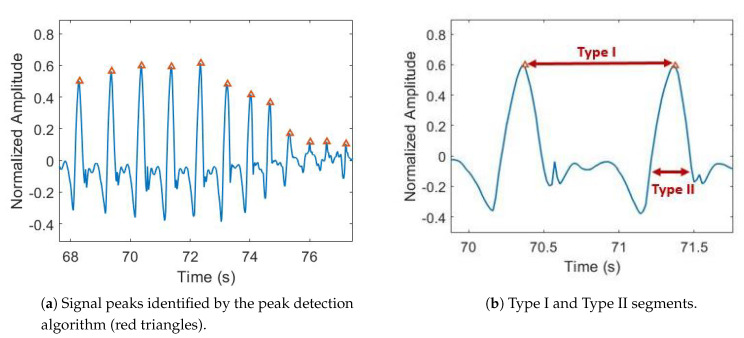
Peak detection and signal segmentation.

In order to get information about the traits and qualities of each step accomplished by the patient, we performed a step-to-step data segmentation. To this aim, we considered signal peaks as anchor points for segmentation; indeed, points in which the angular velocity reaches the maximum are known to represent mid-swing phase in gait analysis [39,40]. This procedure was recognized to ease the step detection and limit detection errors. In more detail, we kept signals from right and left leg separated, and we took into account signal peaks with an amplitude ≥20% of the maximum value, at least 350 ms apart. The amplitude threshold was heuristically selected in order to catch both normal and anomalous steps such as those preceding FOG. The temporal threshold was set to avoid duplicated peak detection during normal gait. In Figure 3a, an example of the outcome of our peak detection algorithm is reported.

Once having identified signal peaks, we performed two data segmentation tasks in order to arrange data frames for subsequent feature extraction. Type I segmentation catches data between two subsequent peaks (i.e., the current and the previous one), whereas Type II segmentation encompasses the positive portion of data inside the current peak (Figure 3b). Type I segments yield frequency information, while range of motion and movement intensity can be computed using the Type II segmentation.

As our goal was to catch walking pattern degradation preceding FOG events, the features to be extracted from inertial data needed to have the ability to represent subtle details of each step. The selected features in both time and frequency domains are reported in Table 3. Some of them, e.g., standard deviation, range and root mean square, are self-explanatory. In the following we provide descriptions of those features requiring some comments.

*Angular jerk.* It represents the rate of variation of the angular acceleration, defined as $\frac{1}{2}\cdot \int \ddot{\omega_{x}^{2}}\cdot dt$, where ${\ddot{\omega }}_{x}$ is the second derivative of the angular velocity around the x-axis.

*Normalized jerk.* It represents the Angular Jerk normalized by the time in which it is computed.

*Stride similarity.* It is computed using the Dynamic Time Warping (DTW) algorithm. It provides a scalar output that is inversely proportional to the similarity between the two input signals. Thus the output represents the similarity between the actual stride and the previous one.

*Step time.* It is computed as the temporal distance between each peak and the previous contralateral peak.

*Stride time.* It is computed as the temporal distance between two subsequent peaks in the signal measured on either the right or the left leg.

*Peak height* and *width*. They are computed respectively as the height (with respect to zero) and half-power width of the positive portion of the signal peak. The former represents the maximum angular velocity reached in each step while the latter is proportional to the swing time.

*Power spectral entropy*. It is the spectral Shannon entropy, computed as −P·log(P+ϵ), where P is the normalized squared amplitude of the signal Fast Fourier Transform (FFT) and ϵ an arbitrarily small value (0.001) ensuring real output values. It represents a measure of the quantity of information carried by the signal spectrum.

*Principal harmonic amplitude* and *frequency.* They are computed from the signal FFT, as the peak value and its corresponding abscissa (frequency).

*Principal harmonic width.* It is obtained as the half-power width of the principal harmonic component.

*Weighted power spectral peak.* It is the product of amplitude and frequency of the principal harmonic.

*Low power frequency.* It represents the ratio between the power in the bandwidth 0–2 Hz and the total signal power.

### 2.4. FOG Detection

We set up a binary supervised classification problem for FOG detection, namely, gait vs FOG. This allowed us to get an insight into the capability of the extracted feature set to discriminate between normal and abnormal gait patterns. In view of the subsequent pre-FOG detection task, the implemented algorithm must be robust and easily interpretable. In this context, decision tree (DT) represents a simple and fast algorithm, providing a straightforward interpretability of its outcome. Nevertheless, said algorithm is known to implement a very sharp margin separating the two classes, thereby increasing the risk of overfitting. On the other hand, support vector machine (SVM) seeks the hyperplane providing the largest margin for separating the two classes and it has been widely employed in similar problems [41,42]. In this work, we combined both models by exploiting DT for feature selection and SVM for classification.

As for DT, features close to the tree root achieve the best classification of the training set. Hence, this algorithm can be used to rank features in decreasing order of relevance. The implemented DT has the following parameters:

- Split criterion: Gini-Simpson diversity index [43].
- Minimum leaf size: 1.
- Maximum number of splits: 15.

In order to identify the best model configuration in terms of selected features and model parameters, we performed a tuning procedure based on mis-classification error minimization in a 10-fold cross validation. The number of features to be selected varied from one to all features, while the regularization parameter of the SVM model ranged between 1 and 20.

We performed a 70/30 training-test procedure in order to avoid overfitting. First, we splitted the feature set into a training (70% of data) and a test set (30% of data). Then, we performed the model optimization using the training set and tested the optimized model on the test set. In Algorithm 1 we report the entire procedure for training, validation and testing of the model.

Moreover, in order to ensure subject independence and to achieve results representative of more realistic working conditions, we performed a Leave-One-Subject-Out (LOSO) validation. It consists of training the model with data from all patients except one, which is used for testing. For each performed validation/test, we evaluated the classification performance employing the metrics reported in Section 2.6. We performed training, validation and test on data related to patients on and off therapy separately. Then we compared the results obtained in the two conditions. Finally, we trained the final model configuration with data related to patients on (off) therapy and tested on off (on) data. Thus we compared the performance obtained in the two testing conditions.
**Algorithm 1** Algorithm for model optimization, validation and test performance evaluation1:**procedure**Performance(Data)2: 3:    **for** k←1 to 20 **do**                            ▹ Perform 20 times train-test procedure4: 5:        [trainingSet,testSet]←split(data,0.3)          ▹ Split data into 70% trainingSet and 30% testSet6: 7:        featureSet←DT(trainingSet)             ▹ Sort features according to Decision Tree algorithm8: 9:        **for** f←1 to length(featureSet) **do**            ▹ Numer of selected features from 1 to the full set10: 11:           **for** c←1 to 20 **do**                         ▹ Regularization cost from 1 to 2012: 13:               error(f,c)← loss(10-fold cv(trainingSet,f,c))         ▹ Perform 10-fold cross validation14: 15:           **end for**16: 17:        **end for**18: 19:        f,c←min(error)                       ▹ Select the best f and c combination20: 21:        Performance(k)←test(model,f,c,testSet)          ▹ Compute performance on testSet22: 23:    **end for**24: 25:    **return** Performance                        ▹ Return performance for each split26: 27:**end procedure**

### 2.5. Pre-FOG Detection

**Figure 4:**
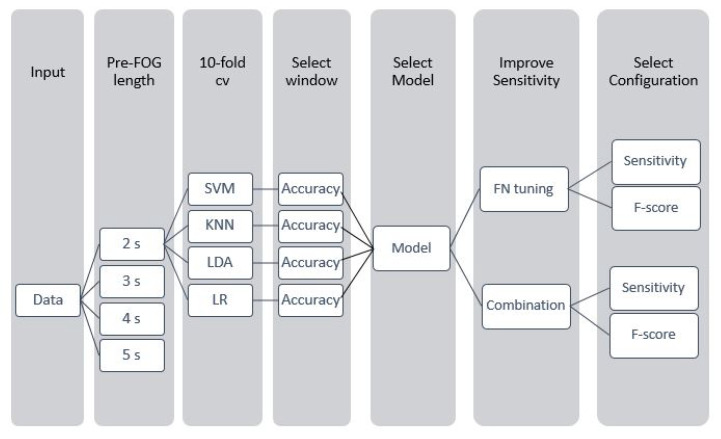
Workflow of the procedure used for the identification of the final model configuration.

As for pre-FOG detection, i.e., capturing typical degradation of gait pattern preceding FOG episodes, we implemented a binary classification problem to differentiate between gait and pre-FOG. In Figure 4 we describe the steps employed to select the final model configuration, which is used for pre-FOG identification.

First of all, as the pre-FOG window length cannot be determined *a-priori*, we took into consideration different window lengths in the range 2–5 s. For each value we labeled the corresponding gait data as belonging to the pre-FOG class. As the identification of the most suitable classification algorithm for this task is not straightforward, we tested the model implemented for FOG detection and different models, namely, k-nearest neighbor (kNN), linear discriminant analysis (LDA) and logistic regression (LR). In order to jointly identify the most suitable window length and classification model, we performed 10-fold cross validation for each model-window length pair and computed accuracy. For each classification algorithm, we optimized the corresponding hyperparameters employing a Bayesian optimization algorithm. In Table 4 we report the hyperparameters and the corresponding range used for model optimization in the 10-fold cross validation procedure. Once having identified the model providing the best performance, we implemented two approaches for improving the algorithm sensitivity. The first one consists of tuning the false negative cost of the algorithm in the range 0–10, in order to reduce the number of pre-FOG samples that are not recognized by the algorithm. The second approach employs a combination of detection models; data samples are classified as pre-FOG if at least one model yields a pre-FOG decision.

In both cases, we computed sensitivity, accuracy and F-score and selected the configuration providing the best performance. Then, we performed LOSO validation, and computed several classification evaluation metrics, reported in Section 2.6.

Finally, in order to provide interpretability of the implemented algorithm, we assessed feature relevance in discriminating gait steps from those related to pre-FOG. To that end, we addressed those features that had been most frequently selected, i.e., in at least 80% of cases in the LOSO validation, and we computed the Spearman correlation coefficient and the associated *p*-value between those features and the class label, with “0” meaning “gait” and 1 “pre-FOG.” This represents a method for quantifying the extent of statistical dependence between pairs of observations and allows us to understand which feature shows an increase or a reduction in its values for data approaching a FOG event. Specifically, positive (negative) values of the correlation coefficient indicate increasing (decreasing) values of features as approaching FOG. Finally, we computed the latency between the pre-FOG detection and the actual FOG occurrence. We performed the abovementioned analysis on data related to patients on and off therapy separately, and we compared the achieved results. Then, we trained the final model configuration with patients on (off) therapy and tested on patients off (on). Finally we compared the performance obtained in the two testing conditions.

### 2.6. Performance Evaluation

In order to provide an exhaustive performance evaluation of both FOG and pre-FOG detection algorithms, and to compare results from patients on and off therapy, we computed different performance metrics, reported in the following. First, let us define true positives (*TP*) and true negatives (*TN*) as the numbers of correctly identified positive and negative samples, respectively. False positives (*FP*) represent the number of negative samples wrongly classified as positive, while false negatives (*FN*) refer to the number of positive samples wrongly classified as negative.

Sensitivity and specificity (Equation (2)) represent the algorithms’ capability of detecting true positive and negative samples, respectively.

(2) $$Sensitivity=\frac{TP}{TP+FN}\;\;\;\;\;\;\;\;\;\;\;\;\;\;\;\;\;\;\;\;Specificity=\frac{TN}{TN+FP}$$

Positive predictive value (PPV) and negative predictive value (NPV) (Equation (3)) represent the precision in detecting positive and negative samples, respectively.

(3) $$PPV=\frac{TP}{TP+FP}\;\;\;\;\;\;\;\;\;\;\;\;\;\;\;\;\;\;\;\;\;\;NPV=\frac{TN}{TN+FN}$$

Accuracy is an overall performance measure, reporting the percentage of correctly classified samples, and F-score is the harmonic mean of sensitivity and PPV (Equation (4)).

(4) $$Accuracy=\frac{TP+TN}{TP+TN+FP+FN}\;\;\;\;\;\;\;\;\;\;\;F-score=2\cdot \frac{Sensitivity\cdot PPV}{Sensitivity+PPV}$$

Yuden index summarizes the performance of the test, taking into consideration both sensitivity and specificity (Equation (5)).

(5) YudenIndex=Sensitivity+Specificity−1

In Section 3 we expressed all the reported performance metrics as percentages. Finally, the receiver operating characteristic (ROC) curve shows the relationship between the true positive rate (i.e., sensitivity) and the false positive rate (computed as 100—specificity) as the classification threshold varies, allowing to visualize the performance of the classification model. The area under the curve (AUC) is an overall measure of correct classification, aggregating measures of performance across all possible classification thresholds.

## 3. Results

The offline clinical assessment of video-recordings by two independent neurologists, experts in movement disorders, identified 41 FOG episodes in PD patients on therapy and 54 FOG episodes in those off therapy. All episodes were used for the FOG detection task. On the other hand, 6 and 10 episodes were excluded for pre-FOG analysis, for patients on and off therapy respectively, as they occurred during gait initiation task, i.e., during the transition between standing up and start walking.

### 3.1. FOG Detection

Table 5 summarizes the algorithm performance, in terms of sensitivity, specificity, accuracy, PPV, NPV, F-score and Yuden index, in detecting FOG episodes in PD patients both on and off therapy.

All the performance metrics exhibited a slight decrease moving from 10-fold validation to 70-30 training-test and LOSO validation, due to a progressively larger portion of data used as test set. The LOSO validation is very close to actual working conditions. It achieved high sensitivity, accuracy and F-score (Table 5), always larger than 90%. Additionally, training/test procedure demonstrated the absence of model over-fitting, as evident from the high performance obtained. When comparing patients on and off therapy, the algorithm achieved similar values of sensitivity, accuracy and F-score in the detection of FOG with LOSO, thereby not showing significant performance differences with respect to L-dopa intake. However, the algorithm showed lower specificity in patients off than those on therapy due to an increased number of false positives after L-dopa withdrawal.

Table 6 reports the algorithm performance in FOG detection after training with data recorded from patients on therapy and tested on patients off therapy, and vice versa.

It can be appreciated that, in general, and especially for sensitivity and Yuden index, the algorithm yielded higher performance in detecting FOG when trained with data from patients off therapy.

### 3.2. Pre-FOG Detection

Table 7 summarizes the accuracy of different ML classifiers in identifying pre-FOG periods in patients on and off therapy, also considering various pre-FOG window lengths (from 2 to 5 s).

All the models exhibited a progressive reduction of accuracy in pre-FOG recognition with the increase of the window length. Indeed, a mean accuracy impairment of about 14% and 10% for patients on and off therapy respectively, was observed when doubling the pre-FOG window length. Overall, the accuracy in detecting pre-FOG in patients off therapy was higher than in patients on therapy. SVM and LDA classifiers provided the best performance in terms of accuracy, with sensitivities of 68.4% and 66.2% respectively.

**Figure 5:**
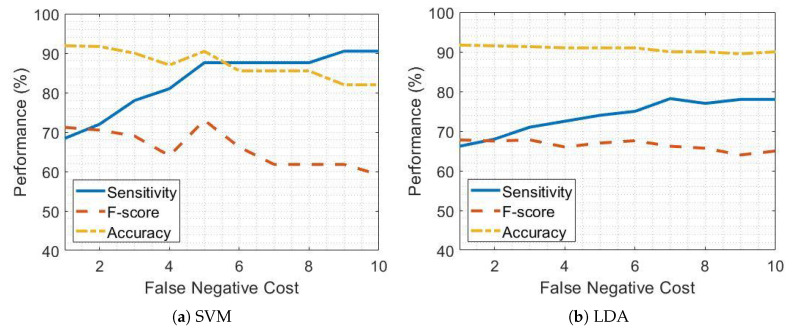
False negative tuning in PD patients receiving dopaminergic therapy (on) for support vector machine (SVM) and linear discriminant analysis (LDA) classifiers.

**Figure 6:**
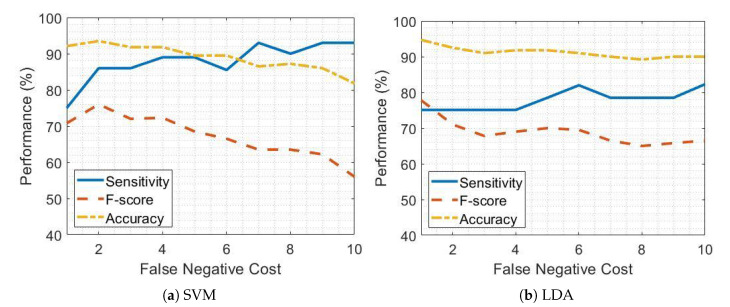
False negative tuning in PD patients not receiving dopaminergic therapy (OFF) for support vector machine (SVM) and linear discriminant analysis (LDA) classifiers.

Figure 5 and Figure 6 report the sensitivity, accuracy and F-score of the SVM and LDA classifiers when detecting pre-FOG periods in patients on and off therapy respectively and considering different FN cost values.

As the FN cost increases, the sensitivity improves but, in turn, the accuracy and F-score get worse due to the occurrence of false positives. This impacts on all the performance evaluation metrics other than sensitivity. Figure 5a and Figure 6a show that, considering SVM classifier and setting an FN cost equal to 5 leads to a sensitivity of 87.5% and 89.2% in patients on and off therapy respectively, while maintaining high values of accuracy and F-score. Conversely, the increase in sensitivity by using the LDA classifier is less satisfactory than for SVM, as shown by Figure 5b and Figure 6b.

Table 8 reports the sensitivity, accuracy and F-score in pre-FOG detection by using the SVM and LDA classifiers separately, with and without FN cost optimization, and the combination of SVM and LDA classifiers in PD patients on and off therapy.

The combination of SVM and LDA classifiers led to increased sensitivity in pre-FOG detection both in patients on and off therapy compared to separate performance of SVM and LDA classifiers. The FN costs equal to 7 and 6 for the LDA classifier (Figure 5b and Figure 6b) exhibited satisfactory performance in patients on and off therapy, respectively. The SVM classifier with an FN cost equal to five achieved the highest performance in pre-FOG detection, mainly in terms of sensitivity, with comparable values of accuracy, for patients both on and off therapy (Figure 5a and Figure 6a).

Table 9 summarizes the sensitivity, specificity, accuracy, PPV, NPV, F-score and Yuden index of the pre-FOG classification algorithm in a LOSO validation in PD patients on and off therapy.

When comparing patients on and off therapy, the classification algorithm demonstrated a different performance in the recognition of pre-FOG periods, with the highest values of sensitivity, specificity, accuracy, F-score and Yuden index after L-dopa withdrawal (i.e., in patients off rather than those on therapy). Moreover, the implemented models detected pre-FOG periods with different latencies in patients off and on therapy. More in detail, when considering patients on therapy, pre-FOG periods were recognized 4 ± 1.1 steps before FOG occurrence. Conversely, pre-FOG periods were recognized 6 ± 1.3 steps before FOG occurrence in patients off therapy. This is probably due to the fact that the pace degradation pattern in off therapy patients is better represented than in on, where it is (partially) corrected by the L-dopa.

**Figure 7:**
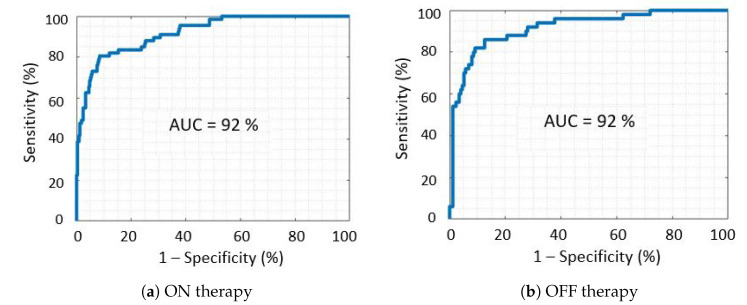
Receiver operating characteristic curves of the final classification model, for patients receiving (ON) and not receiving (OFF) dopaminergic therapy.

Figure 7a,b report the ROC curve of the SVM classifier, in patients on and off therapy, respectively. ROC curves show a similar pattern in both conditions and the AUC value is identical. For specificity values over 80%, slightly higher values of sensitivity can be observed for patients off therapy, compared to those on therapy.

Table 10 reports the algorithm performance in pre-FOG detection after training with data recorded from patients on therapy and then tested on data from those off therapy, and vice versa.

As evident, the different tests on patients on and off therapy led to opposite results both in terms of sensitivity and specificity in the detection of pre-FOG periods. More in detail, the algorithm training with data from patients on therapy and testing on data from patients off therapy showed significantly higher sensitivity and lower specificity than the algorithm training with data from patients off therapy and testing on data from patients on therapy. Indeed, in this latter case, sensitivity was severely impaired and achieved values less than 60%.

**Figure 8:**
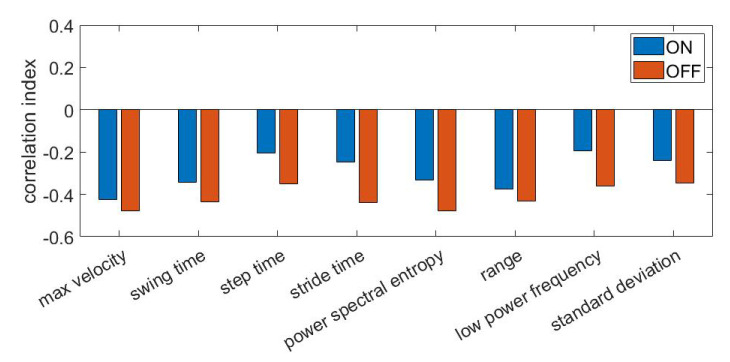
Spearman correlation coefficient between selected features and class label (i.e., 0 and 1 for gait and pre-FOG respectively). A negative correlation denotes decreasing values of features during pre-FOG. ON: receiving dopaminergic therapy; OFF: not receiving dopaminergic therapy.

Finally, Figure 8 shows the Spearman correlation coefficient, computed for the most frequently selected features in patients on and off therapy during pre-FOG periods. All corresponding *p*-values were found to be <0.001.

## 4. Discussion

In this section, we discuss results regarding FOG detection and FOG prediction emphasizing the influence of dopaminergic therapy on the pace pattern degradation and the algorithm performance. To provide an exhaustive performance evaluation of ML algorithms, we computed several metrics for different validation and test methods, and we considered the impact of various pre-FOG window lengths on FOG prediction.

Concerning FOG detection, our algorithm yielded high performance in the recognition of FOG episodes, comparable with those previously described [44,45]. Additionally, in line with previous research [14], our algorithm detected FOG episodes in PD patients on and off therapy with a similar sensitivity, thereby suggesting that L-dopa does not significantly change FOG-related features, but only impacts on the frequency and duration of FOG episodes. Despite comparable sensitivity, our algorithm recognized FOG episodes with lower specificity in patients off than those on therapy. This could reflect an increased number of false positives owing to the difficulty in differentiating abnormal spatial–temporal gait parameters, which are prominent in patients off therapy, from FOG episodes. Finally, training the algorithm in patients off therapy led to better performance in the detection of FOG episodes in patients on therapy than vice-versa. This likely reflects the increased frequency and duration of FOG episodes in patients off than those on therapy, thus providing a larger amount of data for training [14].

Regarding the pre-FOG detection, our classifier achieved performance in line with recent works, confirming the possibility to predict FOG in PD patients by using ML algorithms [32,33]. We also found that the length of the pre-FOG window (i.e., 2, 3, 4, 5 s) crucially affected the accuracy of pre-FOG recognition: the longer the pre-FOG window, the less the prediction accuracy. In line with our hypothesis and previous observations [15], this finding suggests that the walking pattern degradation that commonly precedes FOG, becomes increasingly evident as the FOG episode approaches. Accordingly, short pre-FOG window lengths (i.e., 2–3 s) should be used to improve the overall accuracy of FOG prediction in PD. In detail, during pre-FOG periods in PD patients, the leg movement slows down (i.e., decrease in range, standard deviation and max velocity), steps become faster and shorter (i.e., decrease in step, stride time and swing time), the frequency content of strides becomes more variable (i.e., decrease in power spectral entropy value) and the stride frequency content exhibits a shift towards high frequencies (i.e., decrease in low power frequency).

When assessing the effects of L-dopa on ML performance, we found that the accuracy in pre-FOG detection was higher in patients off than those on therapy, fully in line with our initial hypothesis that the L-dopa impacts on FOG prediction, since pace degradation pattern in on therapy is partially corrected by the L-dopa itself. Further supporting the influence of L-dopa on FOG prediction, our algorithm recognized pre-FOG periods earlier in patients off therapy than in those on therapy (6 ± 1.3 and 4 ± 1.1 steps before FOG occurrence, respectively). Additionally, training the algorithm with data from patients on and off therapy significantly changed the ability to detect pre-FOG. These findings agree with the observation that L-dopa improves spatial–temporal gait parameters outside FOG episodes [13,14]. Accordingly, L-dopa changes the typical degradation pattern preceding FOG episodes by attenuating pre-FOG periods in patients on therapy. Overall, our findings support the need to train ecological ML algorithms for FOG prediction in PD by specifically considering patients’ state of therapy.

When considering the present findings, a few limitations should be taken into account. First, although the patients were selected on the basis of rigorous clinical criteria, confering homogeneity to the cohort, this latter consisted in a limited number of subjects. Accordingly, to further increase the statistical significance of our results, future studies should enrol a larger sample of patients. Second, we did not examine the possible impact of specific FOG phenotypes (i.e., trembling, akinetic, shuffling) on FOG prediction by ML algorithms [24]. Future studies should therefore clarify whether the typical degradation pattern of gait preceding FOG episodes is related to specific FOG phenotypes. Finally, data eventually collected with an increased number of 3D inertial sensor units could allow one to improve the pre-FOG window length while maintaining high accuracy values, enabling the adoption of protective or preventive strategies for FOG in PD patients. However, this approach would require the introduction of new synchronization solutions, as those recently proposed in [46], where a time coordination method based on a master/slave architecture was presented. This kind of solution would also prevent temporal shift between different units in long-time monitoring.

## 5. Conclusions and Future Work

In this work, we used wearable devices integrating inertial sensors and machine learning to detect and predict the occurrence of FOG episodes in Parkinson’s disease. A cohort of 11 selected PD patients was equipped with inertial sensors placed on the shins, and asked to perform a TUG test. We compared the ML performance in the detection of pre-FOG periods in patients on and off dopaminergic therapy. We used a single angular velocity signal from sensors and implemented a step-to-step segmentation process, extracting features from each step. No assumptions regarding the time frame in which the degradation manifests was made; thus we performed the analysis by employing different durations of the pre-FOG window (finally fixed at 2 s). We employed a wrapper approach for feature selection and optimized different ML classifiers in order to catch both FOG and pre-FOG episodes. The implemented FOG detection algorithm achieved excellent accuracy in LOSO validation, in patients both on and off therapy. As for FOG prediction, the implemented classification algorithm achieved 84.1% (85.5%) sensitivity, 85.9% (86.3%) specificity and 85.5% (86.1%) accuracy in LOSO validation, in patients on (off) therapy. When training the classification model with data from on (off) patients and testing on patients off (on), we found 84.0% (56.6%) sensitivity, 88.3% (92.5%) specificity and 87.4% (86.3%) accuracy. It is remarkable that, for the first time, we demonstrated that when predicting FOG in PD through ML algorithms, it is relevant to consider patients’ state of therapy with L-dopa. Indeed, by improving the spatial–temporal parameters of gait outside FOG episodes, L-dopa likely attenuates pre-FOG periods in patients on therapy, thereby affecting the ability to predict FOG through ML algorithms, and in particular, strongly degrading the sensitivity.

## Figures and Tables

**Table 1:** Demographic and clinical features of patients enrolled in the present study (mean ± standard deviation). FOG: Freezing of Gait; H&Y: Hoehn and Yahr.

# Patients (Male)	Age (Years)	Disease Duration (Years)	FOG Duration (Years)	H&Y
11 (7)	73 ± 7	10.5 ± 7	6.7 ± 1.6	2.7 ± 1

**Table 2:** Standardized scales and scores of patients enrolled in the present study (mean ± standard deviation). BAI: Beck anxiety inventory; FAB: frontal assessment battery; FOG-Q: freezing of gait questionnaire; HAM-D: Hamilton depression rating scale; LEDD: L-dopa equivalent daily dose; MDS-UPDRS-III: Movement Disorder Society—unified Parkinson’s disease rating scale, part III; MMSE: mini mental state examination; OFF: not receiving dopaminergic therapy; ON: receiving dopaminergic therapy.

MDS-UPDRS-III ON (OFF)	FOG-Q	MMSE	FAB	HAM-D	BAI	LEDD
37.9 ± 15.1 (44.5 ± 16.9)	18.6 ± 2.9	28.3 ± 2.1	14.4 ± 2.8	17 ± 7.8	16.5 ± 13	741 ± 272

**Table 3:** List of extracted features, along with the segmentation type employed.

Domain	Feature	Segmentation
**Time**	Standard Deviation	Type I
	Range	Type I
	Root Mean Square	Type I
	Angular Jerk	Type II
	Normalized Jerk	Type II
	Stride Similarity	Type I
	Step Time	Type II
	Stride Time	Type I
	Peak height	Type II
	Peak width	Type II
**Frequency**	Power Spectral Entropy	Type I
	Principal Harmonic Frequency	Type I
	Principal Harmonic Amplitude	Type I
	Principal Harmonic Width	Type I
	Weighted Power Spectral Frequency	Type I
	Low Power Frequency	Type I

**Table 4:** Parameters employed for model optimization, along with the corresponding range.

Model	Parameter	Range
SVM	kernel function	linear, quadratic, gaussian
	kernel scale	0.001–100
	box-constraint	0.01–100
kNN	number of neighbors	1–50
	distance metric	euclidean, manhattan
	distance weight	equal, inverse, squared inverse
LDA	Gamma	0.01–1
	Delta	0.01–100
LR	Lambda	0.01–100

**Table 5:** Algorithm performance in FOG detection in Parkinson disease patients receiving (ON) and not receiving (OFF) dopaminergic therapy. cv: cross-validation; NPV: negative predictive value; PPV: positive predictive value.

Evaluation Metric	Condition	10-Fold cv	70-30 Training-Test	Leave-One-Subject-Out
Sensitivity (%)	ON	95.9	93.9	93.7
	OFF	97.1	94.9	93.9
Specificity (%)	ON	95.4	94.2	91.8
	OFF	93.5	90.6	85.0
Accuracy (%)	ON	95.5	94.1	92.6
	OFF	96.3	93.1	92.0
PPV (%)	ON	95.3	93.9	91.7
	OFF	94.2	93.5	86.8
NPV (%)	ON	96.2	94.1	93.8
	OFF	95.7	92.7	91.4
F-score (%)	ON	95.6	93.9	92.7
	OFF	95.6	94.2	90.2
Yuden Index (%)	ON	91.3	88.1	85.5
	OFF	90.6	85.5	78.9

**Table 6:** The algorithm’s performance in FOG detection after training with Parkinson’s disease patients receiving dopaminergic therapy (ON) and testing on PD patients not receiving dopaminergic therapy (OFF), and vice versa. NPV: negative predictive value; PPV: positive predictive value.

Training Set	Test Set	Sensitivity	Specificity	Accuracy	PPV	NPV	F-Score	Yuden Index
ON	OFF	88.0 %	90.3 %	89.0 %	91.9 %	85.8 %	89.9 %	78.3 %
OFF	ON	96.2 %	89.0 %	92.6 %	89.4 %	96.1 %	92.7 %	85.2 %

**Table 7:** Accuracy (%) of different classifiers in pre-FOG recognition by considering various pre-FOG window lengths. kNN: k-nearest neighbor; LDA: linear discriminant analysis; LR: linear regression; SVM: support vector machine.

Window Length (s)	SVM		kNN		LDA		LR	
	ON	OFF	ON	OFF	ON	OFF	ON	OFF
**2**	91.3	92.1	84.7	89.8	91.7	94.7	89.0	90.6
**3**	86.1	88.7	80.2	84.7	85.6	86.4	84.4	85.2
**4**	77.8	84.6	69.4	80.4	78.6	82.6	75.2	81.8
**5**	64.9	74.6	58.9	79.3	65.8	75.4	44.9	71.1

**Table 8:** Performances of support vector machine (SVM) and linear discriminant analysis (LDA) classifiers, separately and in combination, with and without optimized false negative cost, in pre-FOG detection in PD patients receiving (ON) and not receiving (OFF) dopaminergic therapy.

Performance	Condition	SVM	LDA	SVM + LDA	SVM (Optimized Cost)	LDA (Optimized Cost)
Sensitivity (%)	ON	68.4	66.2	72.1	87.5	78.2
	OFF	75.0	75.1	79.2	89.2	82.2
Accuracy (%)	ON	91.8	91.7	91.7	90.2	90.1
	OFF	92.1	94.7	92.9	89.4	91.0
F-score (%)	ON	71.2	67.8	66.7	72.3	66.0
	OFF	71.9	77.8	75.7	68.1	69.8

**Table 9:** Performance of the pre-FOG classification algorithm in the leave-one-subject-out validation in patients receiving (ON) and not receiving (OFF) dopaminergic therapy.

Therapy	Sensitivity	Specificity	Accuracy	PPV	NPV	F-Score	Yuden Index
ON	84.1 %	85.9 %	85.5 %	65.1 %	93.5 %	73.4 %	70 %
OFF	85.5 %	86.3 %	86.1 %	66.2 %	93.0 %	74.6 %	71.1 %

**Table 10:** The algorithm’s performance in pre-FOG detection after training with Parkinson’s disease patients receiving dopaminergic therapy (ON) and testing on PD patients not receiving dopaminergic therapy (OFF), and vice versa.

Training Set	Test Set	Sensitivity	Specificity	Accuracy	PPV	NPV	F-Score	Yuden Index
ON	OFF	84.0 %	88.3 %	87.4 %	66.7 %	95.2 %	74.4 %	72.3 %
OFF	ON	56.6 %	92.5 %	86.3 %	77.9 %	88.2 %	65.6 %	49.1 %

## Data Availability

The data presented in this study are available on request from the corresponding author.

## Abbreviations

The following abbreviations are used in this manuscript:

ADL	Activities of Daily Living
AUC	Area Under the Curve
DT	Decision Tree
DTW	Dynamic Time Warping
FFT	Fast Fourier Transform
FOG	Freezing of Gait
FN	False Negatives
FP	False Positives
IMU	Inertial Measurement Unit
kNN	k-Nearest Neighbor
LDA	Linear Discriminant Analysis
L-DOPA	Levodopa
LOSO	Leave-One-Subject-Out
LR	Logistic Regression
ML	Machine Learning
NPV	Negative Predictive Value
PD	Parkinson’s Disease
PPV	Positive Predictive Value
ROC	Receiver Operating Characteristics
SVM	Support Vector Machine
TP	True Positives
TN	True Negatives
TUG	Timed Up and GO
